# Bio-Inspired Gaze and Neural Command Fusion for Assistive Smartphone Interaction

**DOI:** 10.3390/biomimetics11070514

**Published:** 2026-07-22

**Authors:** Marius-Valentin Drăgoi, Ionuț Nisipeanu, Iuliana Marin, Cozmin Adrian Cristoiu, Cătălin-George Alexe

**Affiliations:** 1 Faculty of Industrial Engineering and Robotics, National University of Science and Technology POLITEHNICA Bucharest, 060042 Bucharest, Romania; marius.dragoi@upb.ro; 2 Faculty of Engineering in Foreign Languages, National University of Science and Technology POLITEHNICA Bucharest, 060042 Bucharest, Romania; ionut.nisipeanu@stud.fils.upb.ro (I.N.); iuliana.marin@upb.ro (I.M.); 3 Department of Entrepreneurship and Management, Faculty of Entrepreneurship Business Engineering and Management, National University of Science and Technology POLITEHNICA Bucharest, 060042 Bucharest, Romania; catalin.alexe@upb.ro

**Keywords:** gaze tracking, brain–computer interface, assistive smartphone interaction, android accessibility, EEG commands, mobile accessibility, FastAPI, hands-free control

## Abstract

This paper presents an assistive smartphone interaction system that combines mobile gaze tracking with EEG-based BCI commands. The Android application estimates the user’s gaze with the front camera, MediaPipe facial landmarks, a TinyTrackerS TFLite model, temporal smoothing, and a calibrated mapping from model output to screen coordinates. The gaze point is used to locate the intended screen area, while the BCI layer uses Emotiv Cortex commands for click, scroll, and back actions. A FastAPI and MongoDB backend manages profiles, calibration data, validation reports, runtime data, and WebSocket control events. Android Accessibility is used to execute the selected actions, with raw tap fallback when needed. The system was tested with 36 student volunteers during a short Patient Assist task. In the evaluation, 33 out of 36 gaze mappings were promoted to the active profile. The average static mean error was 390.02 px, and the average static p95 error was 789.09 px. BCI command success was 89.58% for click, 72.22% for scroll, and 77.78% for back. The Android layer acknowledged 228 out of 234 accepted control events. The average usability score was 4.21 out of 5.

## 1. Introduction

Smartphones are now used for communication, access to information, entertainment, social participation, and everyday digital services. However, most smartphone interfaces are still designed around touch gestures, stable hand control, and precise finger movements. For users with severe motor impairments, upper-limb limitations, paralysis, or reduced voluntary movement, this interaction model can become difficult or impossible to use independently. Current mobile accessibility tools, such as voice access, switch control, scanning interfaces, screen readers, and magnification, are useful in many situations, but they do not fully solve all interaction needs. Voice may be affected by fatigue, speech impairment, or environmental noise, while switch-based interaction may be slow and tiring for tasks that require repeated selection [1,2,3].

Brain–computer interfaces (BCI) have been studied as a possible way to support users who cannot rely on standard motor pathways. Recent reviews and application-oriented studies show that non-invasive BCI systems can be used for communication, device control, home automation, rehabilitation, and assistive interaction [4,5,6,7]. Electroencephalogram (EEG)-based BCI systems are especially relevant for practical prototypes because they are safer and easier to deploy than invasive systems, although they remain sensitive to user-specific signal variability, fatigue, headset contact quality, and training consistency [8,9]. Recent BCI-based assistive systems have shown that mental commands can be converted into practical actions when the task is clearly defined and the command pipeline includes suitable filtering and validation [10,11].

Gaze tracking offers a complementary input channel because the eyes naturally indicate the user’s area of attention. In assistive interaction, gaze can be used to identify the intended screen region without requiring hand movement. Recent studies on gaze-based interfaces have shown the relevance of eye tracking for accessibility, communication, virtual interaction, and human–computer interaction [12,13]. Mobile gaze tracking has also become more realistic because current smartphones include front-facing cameras and enough processing power for lightweight computer vision pipelines [14]. Compact gaze-estimation models, such as TinyTracker, further support this direction because they are designed for low-power and edge-oriented gaze estimation [15].

Still, gaze alone is not always sufficient for reliable interaction. Looking at a button does not necessarily mean that the user wants to activate it. This limitation is often discussed as the “Midas touch” problem in gaze-based interaction. One way to reduce this problem is to separate spatial selection from intentional confirmation. Recent work has explored the use of neural activity as a confirmation trigger for gaze-based interfaces, showing that eye tracking and BCI can be combined to support more deliberate hands-free selection [16]. Hybrid EEG and eye-tracking interaction has also been investigated in immersive environments, where gaze can localize the target and EEG can contribute to hands-free command execution [17].

The system presented in this paper follows this hybrid direction but applies it to assistive smartphone control. The implemented architecture combines gaze-based target localization with BCI-based command triggering. The gaze channel answers the question “where?”, while the BCI channel answers the question “when?”. In practical terms, the user looks toward an interface target, and trained neural commands trigger actions such as click, scroll, and back. This interaction principle is bio-inspired because it follows the natural relation between visual attention, user intention, and action execution.

The technical implementation is based on an Android application, an EEG-based BCI layer, an Android Accessibility execution service, and a backend for profiles, telemetry, calibration, validation, and control routing. The Android gaze pipeline uses the smartphone front camera, MediaPipe facial landmarks, a TinyTrackerS TFLite model, temporal smoothing, and a calibrated polynomial mapping from model output to screen coordinates. The BCI layer uses Emotiv Cortex connectivity and trained mental command profiles. The backend, implemented with FastAPI and MongoDB, manages device profiles, gaze calibration sessions, validation reports, runtime samples, mapping priors, Cortex status, and WebSocket-based control events. The Android Accessibility layer executes semantic interface actions on detected interface targets, with raw tap fallback when no semantic target is available. These elements are implemented in the application described in the project documentation, which also includes the patient-facing application, event logging, retry-based upload, and server-side event deduplication.

The proposed work is related to recent bionic and bio-inspired systems in which biological control principles are translated into technical interfaces. Previous work on bionic prostheses has shown that human movement principles, neural control, and adaptive mechanical design can be combined to improve assistive technologies [18]. More recently, Song et al. demonstrated that continuous neural control of a bionic limb can restore more natural walking patterns in people with amputation, supporting the broader idea that technical systems can benefit from tighter integration with biological control signals [19]. A similar direction is reported in tissue-integrated bionic knee research, where the integration between the human body and the prosthetic system improved versatile legged movement after amputation [20]. This perspective is relevant for the present work because the proposed smartphone interface also uses a biological control channel, namely EEG-based command triggering, as part of an assistive interaction loop.

The system is also connected to recent work on touchless and vision-based interaction. Gesture-controlled local computer-vision platforms show that camera-based input can support accessible and hands-free interaction in digital environments [21]. At the same time, recent hybrid EEG and eye-tracking systems such as NeuroGaze show that gaze and EEG can be combined into a single hands-free control interface, supporting the idea that multimodal biological input can make interaction more flexible than a single input channel alone [22]. Vision-based teleoperation studies further show that camera-only human tracking can be used to control external systems without specialized wearable hardware [23]. Compared with these directions, the present work focuses specifically on assistive smartphone interaction, where the challenge is not only to detect the user’s intention but also to execute reliable selections inside a mobile interface.

A central element of the implemented system is adaptation. Gaze tracking varies with lighting, head pose, phone position, camera characteristics, glasses, and user posture. EEG-based BCI commands vary across users and sessions because each user produces different neural patterns and may train mental commands differently. For this reason, the system includes two practical adaptation loops. The first loop is gaze calibration and validation: the user follows screen targets, the system fits a polynomial gaze-to-screen mapping, validation metrics are computed, and only acceptable mappings are promoted to the active profile. The second loop is BCI training and activation: the user trains or loads a mental command profile, and the backend accepts commands only after confidence, consecutive-detection, and cooldown checks. This design avoids a simple “last calibration wins” strategy and instead uses quality gates before promoting mappings or executing commands.

The patient application is another implemented part of the system. It provides a simplified interface with large tiles for Help, Needs, Read, and Control actions. The layout avoids dense controls and uses large interaction areas to make gaze-based selection easier, even when calibration is not perfect. Sensitive actions can be routed through a confirmation screen, while patient actions are logged locally, uploaded with retry support, and deduplicated on the server using event identifiers. This design makes the system more practical for assistive use because it separates the technical configuration application from the patient-facing interaction layer.

The main contribution of this paper is an implemented hybrid gaze–BCI architecture for assistive smartphone interaction. The contribution is not limited to a single gaze model or a single BCI command but consists of the integration of several working components: Android gaze tracking, calibrated gaze-to-screen mapping, Emotiv Cortex-based command triggering, WebSocket control routing, Android Accessibility execution, profile and validation management, telemetry storage, and a simplified patient application. The prototype is presented as an engineering and feasibility-oriented framework. Further work will focus on multi-user testing, cross-device validation, longitudinal use, and comparison with gaze-only, voice-based, and switch-based accessibility workflows.

## 2. Materials and Methods

The proposed system was developed as an assistive smartphone interaction platform that combines mobile gaze tracking with EEG-based BCI command triggering. The system allows the user to select a smartphone interface target by gaze and to activate it through a trained mental command. The implemented prototype includes an Android patient application, an Android control application, a laptop running the backend and BCI connection services, and an Emotiv EEG headset (Emotiv, Inc., San Francisco, CA, USA).

**Figure 1:**
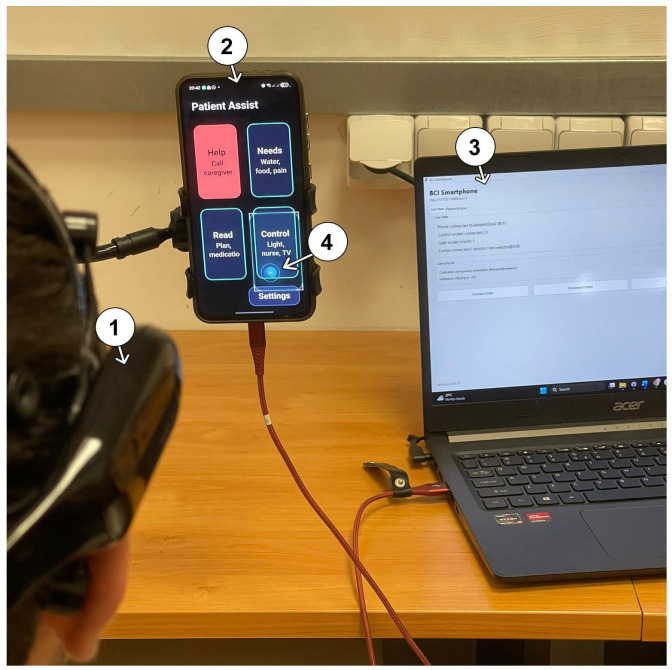
Implemented hybrid gaze–BCI smartphone interaction system: 1. User wearing the Emotiv EEG headset; 2. Android smartphone running the Patient Assist application; 3. Laptop running the BCI Smartphone control view and connected backend/Cortex services; 4. Real-time gaze cursor positioned over a selectable patient interface tile.

Figure 1 shows the implemented system during real-time operation. The user wears the EEG headset while looking at the smartphone screen. The smartphone runs the Patient Assist application, which contains large tiles for basic assistive actions. The laptop runs the BCI Smartphone control view and displays the connection state of the smartphone, control socket, gaze socket, and Cortex session. The gaze cursor can be observed over one of the patient interface targets.

**Figure 2:**
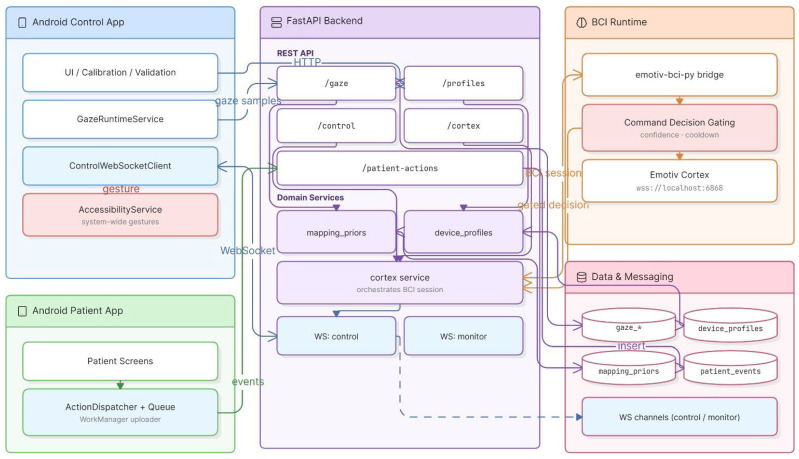
Main software architecture of the implemented hybrid gaze–BCI smartphone interaction system.

The complete software architecture of the implemented system is presented in Figure 2. The architecture contains two Android applications, a FastAPI backend, a BCI runtime environment based on Emotiv Cortex, and a data and messaging layer based on MongoDB and WebSocket communication. The system includes two adaptation loops. The first loop concerns gaze calibration, mapping validation, profile promotion, and runtime gaze tracking. The second loop concerns BCI mental command streaming, decision filtering, and Android control-action execution.

### 2.1. Technology Used

The implemented system uses an Android-based interaction layer, a Python 3.14.6 backend, and an EEG-based BCI command layer. The Android control application manages gaze tracking, calibration, validation, profile access, control-event reception, and control-action execution. A separate Android patient application provides a simple interface designed for assistive interaction.

The gaze component uses the smartphone front camera as the visual input source. Facial landmark detection is performed through MediaPipe-based processing [24]. The detected facial region is provided to the TinyTrackerS TFLite model, which is used as a lightweight gaze-estimation model suitable for mobile operation [15]. In the present system, the TinyTrackerS output is treated as a two-dimensional gaze-related feature. It is not used directly as a final screen coordinate. The final position on the smartphone display is obtained through temporal smoothing and user-specific calibration.

The BCI component uses the Emotiv runtime and the Cortex API workflow [25]. A trained mental command profile is loaded for the active user. During live interaction, mental command events are received by the backend and filtered before being converted into control actions such as click, scroll, or back. The BCI component therefore provides the action trigger, while the gaze component provides the position of the intended target.

The backend is implemented using FastAPI. It manages gaze calibration sessions, gaze validation reports, device profiles, runtime gaze samples, mapping priors, Cortex connectivity, control events, and patient action records. MongoDB is used to store the persistent data produced during system operation. WebSocket communication is used where a real-time connection is required, especially for control-event delivery and monitoring.

The Android execution layer uses AccessibilityService [26]. When the backend sends a control event, the Android control application uses the current gaze position to search for the intended clickable interface target. When a semantic target is found, the requested action is executed on that interface element. When no suitable semantic target is available, a configurable raw tap fallback can be used for click-type actions.

The patient application was designed as a reduced interface for assistive interaction. Its home screen contains large tiles for Help, Needs, Read, and Control. Additional screens allow confirmation of sensitive actions and local inspection of recorded actions. The large target design supports gaze-based selection and reduces the effect of small tracking errors.

### 2.2. Methods

The proposed method is based on the separation of spatial selection and action confirmation. During interaction, gaze is continuously estimated and mapped to a location on the smartphone display. The BCI component remains active in parallel and detects trained mental commands. When a mental command passes the filtering conditions, it is transformed into a control event. Depending on the command class, the Android layer executes click, scroll, or back actions, while the gaze point is used for target selection when a click action is required.

The operation of the system is divided into three connected pipelines. The first pipeline manages gaze calibration, validation, profile promotion, and runtime gaze estimation. The second pipeline manages the connection to Emotiv Cortex, profile activation, mental command streaming, and decision filtering. The third pipeline manages the delivery of the accepted command to Android, the selection of an accessible target, the execution of the requested control action, and the return of an acknowledgement message.

#### 2.2.1. Gaze Tracking, Calibration, and Runtime Mapping

The gaze pipeline starts with a calibration session. The user opens the calibration screen and observes a set of visual targets displayed on the smartphone. During this process, the gaze runtime captures camera frames and facial landmarks. The model output corresponding to each known screen target is collected and transferred to the gaze service.

The implemented application uses a calibrated mapping because the gaze model output cannot be interpreted as a final screen position without adjustment. Differences in user posture, smartphone placement, camera geometry, distance from the screen, and lighting can change the relation between model output and screen location. Calibration creates a mapping that is specific to the active profile and device.

The polynomial mapping is based on the following feature vector:

(1) $$f\left(m_{x},\;m_{y}\right)=\left[1,m_{x},m_{y},m_{x}m_{y},m_{x}^{2},m_{y}^{2}\right],$$

where $m_{x}$ and $m_{y}$ are the two output components provided by the gaze model. The feature vector includes the cross-term $m_{x}m_{y}$. This term was included to capture possible interaction between the horizontal and vertical gaze-model outputs, especially for diagonal or coupled gaze behaviour. Therefore, the implemented mapping already considers both independent quadratic effects and the interaction between the two gaze-model components.

The horizontal and vertical screen coordinates are then estimated as follows:

(2) $$\hat{x}=w_{x}^{T}\;f\left(m_{x},m_{y}\right),$$

(3) $$\hat{y}=w_{y}^{T}\;f\left(m_{x},m_{y}\right).$$

The mapping is fitted using Ridge regression with a regularization parameter of λ = 0.12. The value of λ was selected empirically during prototype tuning and was kept fixed during the reported experiment in order to use the same calibration procedure for all participants. This setting was chosen to provide stable mappings without adding a per-participant optimization step during the short calibration workflow. A grid search or cross-validation procedure was not used in the present feasibility evaluation. Before fitting, the recorded samples are grouped by target position and reduced using robust statistics. This reduces the influence of blinking, temporary tracking errors, or sudden head movement.

After calibration, the system runs two validation stages. The first stage is a static baseline validation, in which the user observes a grid of 25 targets displayed on the smartphone screen. This stage measures the mapping accuracy under stable operating conditions. The second stage is a rotate-phone validation, in which the smartphone is slightly rotated while the user follows the displayed target. This stage is used to verify whether the calibrated mapping remains usable when the device position changes during interaction. The stored metrics include mean screen error, p95 screen error, horizontal and vertical coverage, correlation indicators, and cross-talk indicators. For the experimental sessions reported in this study, a mapping was accepted for live interaction only when the validation mean error was ≤420 px and the validation p95 error was ≤900 px, together with acceptable coverage and correlation values.

The implemented quality thresholds are shown in Table 1.

**Figure 3:**
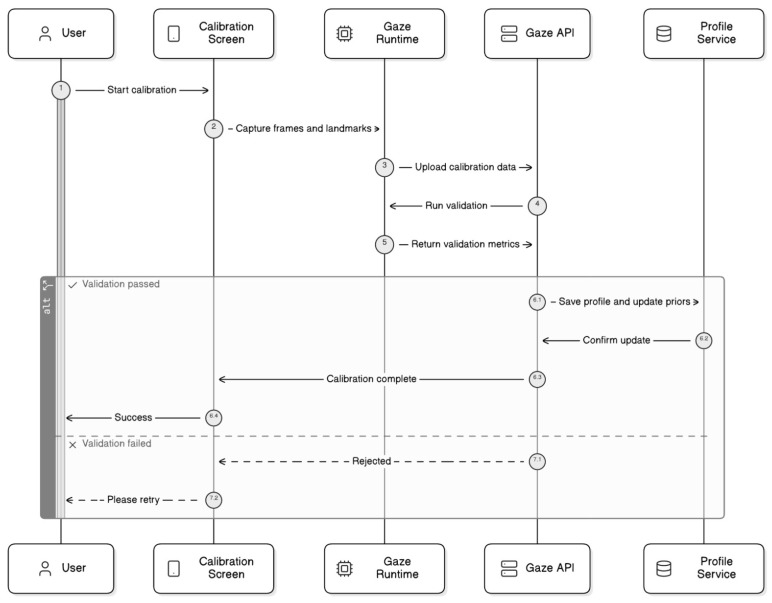
Gaze calibration, validation, profile promotion, and runtime mapping pipeline.

When both validation stages are successfully completed, the profile service saves the new mapping and updates the mapping priors used during later runtime sessions. When either validation stage fails, the mapping is rejected and the user repeats the calibration procedure (see Figure 3).

During normal operation, the gaze runtime processes frames from the smartphone camera and calculates the current screen point. The raw output is stabilized with a One Euro temporal filter before the calibrated mapping is applied. This filter reduces the movement of the gaze cursor when the user is looking at a stable location, while keeping the cursor responsive when the user changes the observed target.

If the system loses valid facial landmarks for several frames, the gaze state is changed to a lost state. In this situation, the system does not continue to issue new screen points until a valid face position is recovered. This avoids the use of unreliable gaze coordinates during target selection.

#### 2.2.2. BCI Profile Activation and Command Filtering

The BCI pipeline is responsible for converting a trained mental command into an accepted system command. The Android interface first requests a BCI connection through the FastAPI endpoint. The backend then starts the Cortex service, which communicates with the BCI client and the Emotiv runtime.

The BCI connection procedure includes an access request, authorization, headset connection, profile loading, and session activation. If these steps are completed successfully, the Android interface is notified that the BCI system is ready. If the authorization or connection fails, an error message is returned and the live command stage is not started.

Before participating in the live test, the user trained three active mental commands in the Emotiv environment. In the implemented system, these commands were mapped to click, scroll, and back actions. The click command was used to activate the currently observed smartphone target, while the scroll and back commands were used to test whether the BCI layer could support additional smartphone navigation actions. This design kept the interaction task simple, but it also tested more than a single confirmation command.

While the system is subscribed to the command stream, the Emotiv runtime forwards raw command events to the BCI client. The Cortex service receives these events and applies a decision gate. The gate uses three conditions: minimum confidence, consecutive detection, and cooldown time. The active command is accepted only when its confidence is at least 0.60, it is detected in three consecutive command samples, and the previous accepted trigger is outside the configured cooldown interval of 800–1000 ms. This decision gate was intentionally conservative. When a command sample was below the confidence threshold, the consecutive counter was reset. This reduced accidental triggers during the short Patient Assist task, but it could also reject a valid command when the BCI stream was briefly interrupted by noise or a temporary confidence drop.

**Figure 4:**
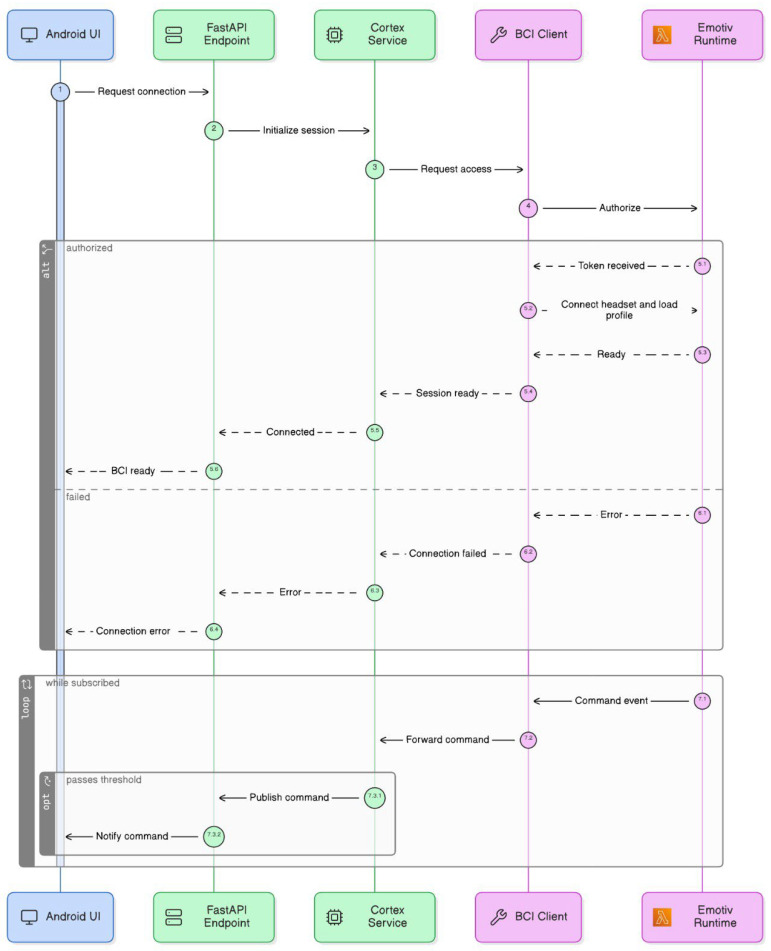
BCI connection, cortex profile activation, mental command streaming, and command-decision filtering pipeline.

These rules are used to reduce accidental activation and repeated clicks. A short neural fluctuation does not immediately create a smartphone action. Only a sufficiently stable command is forwarded by the Cortex service and published through the FastAPI endpoint. The complete connection and command-filtering workflow is presented in Figure 4.

#### 2.2.3. Android Accessibility-Based Command Execution

After a BCI command has been accepted, the backend sends a control event to the connected Android client. The event contains a unique event_id, the selected device identifier, the action type, the source of the action, and the command confidence. In the current implementation, the accepted BCI action is converted into a control request. Click commands use the current gaze point for target selection, while scroll and back commands are executed as navigation actions.

The Android client first logs the received event and checks whether interface targets have already been cached. If cached targets are available, the event can be processed without repeating the complete interface-tree search. If no cached targets are available, the Accessibility service queries the visible user-interface tree and retrieves the available clickable elements.

When more than one clickable target is available near the current gaze position, the service selects the most probable target. The target-selection logic does not depend only on distance to the centre of a button. It also considers whether the gaze point is located inside the target and how close it is to the target boundary. This approach is useful for the patient application because its large neighbouring tiles may be close to each other.

To avoid unstable changes between neighbouring targets, the selected target is supported by a temporal intention mechanism. The mechanism maintains the current likely target over short gaze fluctuations and allows a target change only when the new candidate becomes sufficiently stronger.

If a suitable semantic target is found, Android Accessibility executes the corresponding action on that target. When the semantic target cannot be identified and fallback mode is enabled, the system performs a raw tap at the current gaze position. After the action is completed, an acknowledgement message is returned to the backend. The result is logged, and the target cache can be updated. Events are deduplicated using the event_id value.

**Figure 5:**
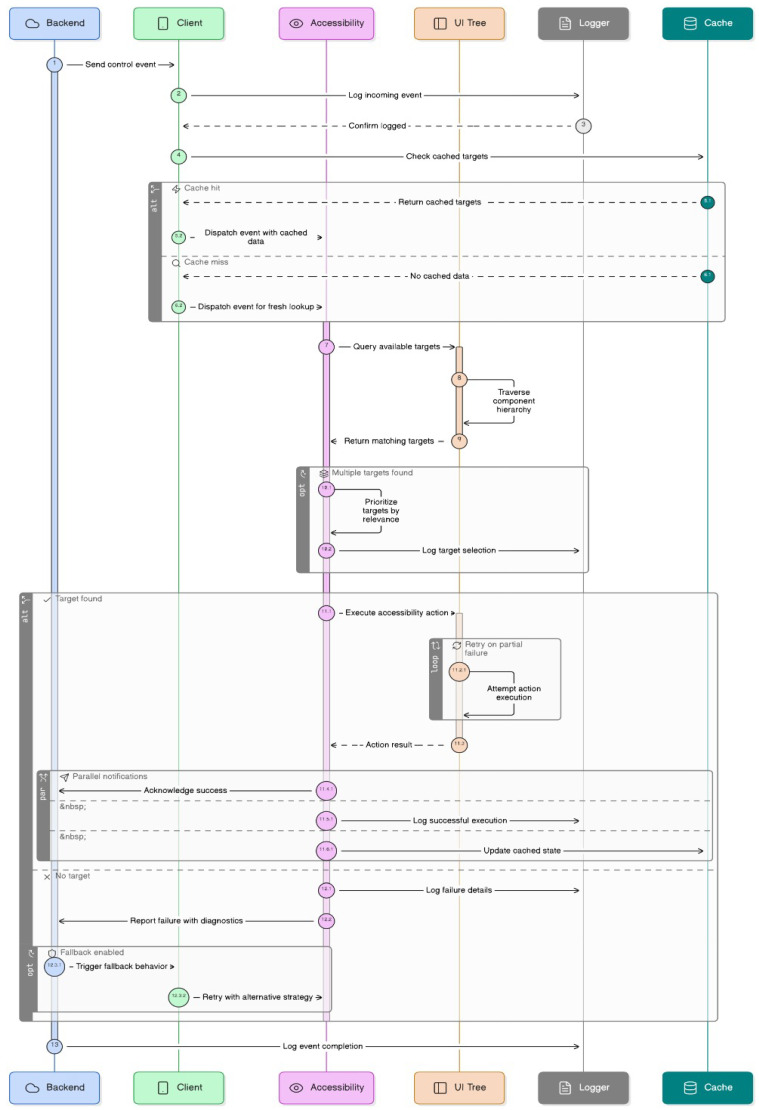
Android Accessibility command execution pipeline, including semantic target selection, raw tap fallback, acknowledgement transmission, and event_id-based deduplication.

The Android execution workflow is presented in Figure 5.

### 2.3. System Implementation

The implemented system contains two Android applications, a FastAPI backend, a local BCI runtime, and a MongoDB-based data storage layer. These components are connected through HTTP/JSON endpoints and WebSocket channels, as shown in Figure 2.

The Android control application is responsible for system configuration and real-time gaze operation. Its main screen provides access to calibration, validation, profiles, system settings, and runtime control. The GazeTrackingManager coordinates the processing of camera frames. The TinyTrackerRunner loads and runs the TinyTrackerS TFLite model. The FaceCropExtractor prepares the facial input region based on MediaPipe landmarks. The GazeSmoother applies temporal filtering, while the GazeToScreenMapping component applies the active calibrated mapping.

Continuous gaze operation is performed through GazeRuntimeService, which runs as a foreground Android service. This allows gaze estimation to continue while the patient application is displayed. The control application also includes ControlWebSocketClient, which receives control commands from the backend, and ProfileRepository, which manages mapping and profile information received from the server.

The Android execution component is implemented through GazeControlAccessibilityService. This service reads the active screen elements exposed through the Android Accessibility tree and selects the intended target based on the current gaze position. The system also includes SemanticTargeting for geometric target selection and TemporalTargetIntentEstimator for stabilizing the selected target over time.

The patient application is the interface used during the assistive interaction task. Its home screen contains the four main areas visible in Figure 1: Help, Needs, Read, and Control. The interface uses large tiles and short labels. The Help section supports predefined assistance requests. The Needs section contains common daily needs. The Read section presents short information pages. The Control section contains simple environmental control actions. For selected sensitive actions, the user is redirected to a confirmation screen before the event is saved.

The patient application stores each completed or cancelled action through its local logging mechanism. The ActionDispatcher component manages the selected action and the confirmation flow. The ActionUploadQueue stores pending events on the device. The ActionUploader and ActionUploadWorker attempt to send the events to the backend and retry the upload if the connection is temporarily unavailable.

The FastAPI backend provides separate endpoint groups for gaze, profiles, control, Cortex communication, and patient actions. The gaze endpoints receive calibration sessions, validation results, and runtime samples. The profile endpoints manage active profiles and mapping priors. The Cortex endpoints manage BCI connection and command publication. The control endpoints route accepted control events to the Android control application. The patient-action endpoints receive and store the actions generated in the simplified patient interface.

MongoDB is used to store six main data groups: gaze calibration sessions, gaze validation reports, gaze runtime samples, device profiles, gaze mapping priors, and patient action events. The backend also uses WebSocket channels for control events sent toward the Android control application and for monitoring events sent toward an operator or caregiver interface.

### 2.4. System Control

The control of the proposed system is based on two simultaneous input streams. The first stream is the continuously updated gaze point. The second stream is the BCI mental command received through Emotiv Cortex. A control action is issued only when the BCI command passes the decision gate. For click commands, the latest valid gaze point is used to determine the smartphone interface target. For scroll and back commands, the accepted command is routed as a navigation action.

In the main workflow, gaze provides the target position, BCI provides the action trigger, and Android Accessibility executes the selected command after the implemented validation checks. The control process is divided into three practical stages: gaze calibration and profile promotion, BCI command filtering, and Android Accessibility-based execution.

The detailed pseudocode for these stages is provided in Appendix A. Algorithm A1 describes gaze calibration, validation, and profile promotion. Algorithm A2 describes the BCI connection and neural command decision gate. Algorithm A3 describes Android Accessibility target selection and control execution.

Each patient action is also recorded by the dedicated application. When the participant selects a tile, the action is stored locally before it is sent to the backend. When an upload is interrupted, the event remains in the local queue and is later resent. The backend checks the unique event identifier and stores each event only once.

### 2.5. Experimental Procedure

The implemented system was evaluated with 36 student volunteers in a controlled indoor environment. The purpose of the evaluation was to test the operation and usability of the hybrid gaze–BCI smartphone interaction system. The evaluation did not involve medical diagnosis, treatment, or clinical intervention.

Each participant completed one individual testing session of approximately 55 min. Before starting the practical procedure, the participant received a short explanation of the system, the testing stages, and the type of data recorded during interaction. Written informed consent was obtained from each participant, and all technical results and questionnaire answers were stored using anonymous participant codes. The consent form stated that participation was voluntary, that the participant could withdraw at any time before data aggregation, and that no names, student identification numbers, images, videos, or medical data would be stored in the research dataset.

The experimental setup included the Emotiv EEG headset, an Android smartphone running the Patient Assist application, and a laptop running the backend and Cortex connection services, as shown in Figure 1. The smartphone was placed on a fixed support in front of the participant, under constant indoor lighting and without strong reflections on the screen or on the participant’s glasses. This setup was selected to remain close to a practical use scenario while reducing avoidable variations during calibration.

At the beginning of the practical procedure, the camera and Android Accessibility permissions were checked, and the foreground gaze runtime service was started. An anonymous user profile was then created or selected for the participant. This profile was used to associate the gaze calibration, validation results, BCI configuration, and interaction records with the same anonymous session.

The gaze calibration stage used sequential visual targets displayed on the smartphone screen. Each participant completed a calibration session using targets distributed over the available screen area. Between 9 and 25 targets were used, depending on the need for an additional calibration pass, and each target was displayed for approximately 1.2 s. The application recorded the gaze-model output for each known target position and fitted the polynomial gaze-to-screen mapping described in Section 2.2.1. If the fitted mapping did not satisfy the quality checks, the calibration was repeated.

After calibration, each participant completed a static baseline validation. In this stage, the participant observed a grid of 25 points displayed on the smartphone screen. The system compared the estimated gaze coordinates with the known target coordinates and calculated the validation metrics. A second validation stage was then performed using the Rotate Phone mode. During this stage, the participant followed the target while the smartphone was slightly rotated, in order to test the robustness of the gaze mapping under small device-position changes.

The calibrated mapping was promoted to the active participant profile only when the validation conditions were satisfied. For the experimental procedure, a mapping was considered suitable for live interaction when the validation mean error was no greater than approximately 420 px and the validation p95 error was no greater than approximately 900 px, together with acceptable coverage and correlation indicators. When these conditions were not reached, the participant repeated the gaze calibration and validation procedure.

After a mapping was accepted, each participant completed a short gaze test on the Patient Assist interface. During this stage, the participant observed several large interface tiles for approximately 1–2 min. This step verified that the large Help, Needs, Read, and Control tiles could be reached reliably using the accepted gaze mapping before the BCI confirmation stage was started.

When a mapping remained unsuitable for persistent profile promotion after recalibration, the participant was still allowed to complete the live interaction task using the latest session-local mapping. During these sessions, semantic-target selection and the permitted raw tap fallback reduced the effect of lower gaze-mapping accuracy. Therefore, mapping promotion indicated suitability for persistent reuse and did not determine eligibility for completing the live task.

The participant then wore the Emotiv EEG headset. The headset position and signal quality were checked before the BCI training session. Each participant trained a neutral state and three active mental commands in the Emotiv environment, corresponding to click, scroll, and back. The trained commands were used as control triggers for click, scroll, and back actions during the live task. Once the trained BCI profile was loaded and the Cortex connection was confirmed, the participant continued to the live hybrid interaction task.

During the live interaction task, each participant followed the same predefined Patient Assist sequence. The Patient App remained open during the complete task. The sequence had a maximum duration of 160 s, corresponding to 2 min and 40 s. Each step had a maximum allowed duration. If the required gaze fixation or BCI command was detected and confirmed earlier, the system moved directly to the next step. If the required action was not completed within the allowed time window, the step was marked as unsuccessful and the sequence continued.

First, the participant fixed the gaze on the Needs tile, which contains options such as water, food, and pain, for up to 10 s. The participant then imagined the click action for up to 20 s, followed by the scroll action for up to 20 s. Next, the participant fixed the gaze on the Tired option for up to 10 s and imagined the click action for up to 20 s. The participant then imagined the back action for up to 20 s in order to return to the previous interface level. After this, the participant fixed the gaze on the Help tile for up to 10 s and imagined the click action for up to 20 s. Finally, the participant fixed the gaze on the Call Caregiver option for up to 10 s and imagined the click action for up to 20 s. The same order and maximum time windows were used for all 36 participants.

For each requested interaction, the system recorded the intended target, the selected target, the execution method, the acknowledgement status, and the response time. The execution method showed whether the action was completed through a semantic Android Accessibility click or through the raw tap fallback. Patient action events were stored using unique event identifiers, allowing repeated uploads to be identified and rejected by the backend.

The researcher filled in a paper observation sheet for each anonymous participant while the system also recorded data automatically. The sheet included calibration status, completed task stages, timeout events, execution methods, acknowledgement outcomes, and short technical observations. The paper observation sheets were used to cross-check the automatically recorded session data and to complete non-critical status fields when a log entry was not available.

At the end of the practical task, each participant completed a short anonymous usability questionnaire. The questionnaire included five-point Likert-scale items related to ease of use, interaction comfort, ease of gaze-based target selection, perceived reliability of the BCI trigger, and overall interest in this type of hands-free interaction.

The testing procedure completed by each participant is presented in Table 2.

The standardized live Patient Assist sequence used during the interaction task is detailed in Table 3.

The live sequence included four gaze-fixation windows and six BCI command-imagery windows. The BCI command-imagery windows contained four click commands, one scroll command, and one back command. Since each time window represented a maximum duration, successful actions could move the participant to the next step before the full 160 s had elapsed.

### 2.6. Evaluation Measures

The evaluation considered the performance of the gaze component, the BCI command component, the Android execution layer, and the usability of the complete interaction system. All measurements were associated only with anonymous participant codes.

The gaze component was evaluated using the results produced during the validation stage. The recorded measures included the validation mean error, the validation p95 error, the horizontal and vertical screen coverage, and the acceptance status of the calibrated mapping. These indicators were used to determine whether the gaze-to-screen mapping was sufficiently stable for the live interaction task.

The mean gaze error was calculated as the average Euclidean distance between the requested validation target and the screen point estimated by the system:

(4) $$E_{mean}=\frac{1}{n}\sum_{i=1}^{n}\sqrt{{\left(x_{i}-{\hat{x}}_{i}\right)}^{2}+{\left(y_{i}-{\hat{y}}_{i}\right)}^{2}}$$

where $x_{i}$ and $y_{i}$ represent the position of the displayed validation target, while ${\hat{x}}_{i}$ and ${\hat{y}}_{i}$ represent the position estimated through the gaze pipeline. The p95 error represented the 95th percentile of the same positional-error values and was used to identify larger errors that may affect target selection.

The BCI component was evaluated using the mental command events received during the live test. The recorded measures included the number of accepted BCI triggers, the number of rejected low-confidence events, and the number of accepted triggers that were converted into control events. A BCI trigger was considered accepted only when it satisfied the confidence threshold, the consecutive-detection condition, and the cooldown rule defined in the implemented command gate.

Because the live task used three mental command classes, the BCI evaluation also considered command-specific success. Click, scroll, and back were analyzed separately. For each command type, the number of expected command periods, accepted command events, rejected command events, and correctly routed actions was recorded. This made it possible to observe whether the click command was easier to execute than the navigation commands.

For each step of the standardized live task, success was defined as completion within the maximum allowed time window. If the expected gaze fixation or BCI command was detected and confirmed before the timeout, the step was counted as successful and the participant advanced to the next step. If the timeout expired before completion, the step was counted as unsuccessful. Therefore, the 160 s live sequence represented the maximum duration of the task, not a fixed waiting time for every participant.

Command-specific success was calculated as follows:

(5) $$A_{command}=\frac{N_{correct\;routed\;commands}}{N_{expected\;commands}}\;\times 100$$

where $N_{correct\;routed\;commands}$ represents the number of accepted BCI commands routed to the correct smartphone action within the allowed time window, and $N_{expected\;commands}$ represents the number of expected commands of the same type in the standardized task.

The timeout rate was calculated as follows:

(6) $$R_{timeout}=\frac{N_{timeout\;steps}}{N_{expected\;steps}}\;\times 100$$

where $N_{timeout\;steps}$ represents the number of gaze-fixation or BCI command steps that were not completed within the maximum allowed time window, and $N_{expected\;steps}$ represents the total number of expected steps in the standardized live task.

The interaction performance of the complete system was evaluated using target-selection accuracy, successful action-execution rate, command-response latency, semantic-click execution rate, raw tap fallback rate, and acknowledgement success rate.

Target-selection accuracy was calculated as follows:

(7) $$A_{target}=\frac{N_{correct\;targets}}{N_{requested\;targets}}\;\times 100$$

where $N_{correct\;targets}$ represents the number of requested interface targets that were correctly activated, and $N_{requested\;targets}$ represents the total number of target-selection tasks completed by the participant.

The successful action-execution rate was calculated as follows:

(8) $$A_{execution}=\frac{N_{acknowledged\;actions}}{N_{accepted\;control\;events}}\;\times 100$$

An action was counted as successfully executed when the Android control application returned an acknowledgement confirming that either a semantic accessibility control or a permitted raw tap fallback had been completed.

Command-response latency was measured only for successful steps. For BCI command steps, latency was measured from the moment when the BCI command passed the decision gate to the moment when the Android application returned the execution acknowledgement. For gaze-fixation steps, completion time was measured from the beginning of the fixation window to the moment when the required target fixation was confirmed.

The semantic-click execution rate was used to determine how often the system was able to activate a meaningful Android Accessibility target instead of applying a direct screen tap.

The semantic-click execution rate was calculated as follows:

(9) $$R_{semantic}=\frac{N_{semantic\;clicks}}{N_{executed\;actions}}\;\times 100$$

The raw tap fallback rate was calculated as follows:

(10) $$R_{fallback}=\frac{N_{raw\;tap\;actions}}{N_{executed\;actions}}\;\times 100$$

The post-test questionnaire was used to assess participant-perceived usability. The questionnaire used a five-point Likert scale, where 1 represented the lowest evaluation and 5 represented the highest evaluation. The analysis considered ease of use, interaction comfort, ease of gaze-based selection, perceived reliability of the BCI trigger, and general interest in hands-free smartphone interaction. For each questionnaire item, the mean score and standard deviation were calculated.

The recorded measures are summarized in Table 4.

## 3. Results

The evaluation was conducted with 36 student volunteers. All participants completed the individual testing protocol described in Section 2.5. The recorded data included gaze calibration and validation results, BCI command outcomes, Android execution status, task completion indicators, response times, and usability questionnaire scores.

The Results section is organized into four parts. First, the gaze calibration and validation results are presented. Second, the BCI command outcomes are reported for click, scroll, and back actions. Third, the complete interaction performance is analyzed using target selection, action execution, timeout, and latency indicators. Finally, the usability questionnaire results are summarized.

### 3.1. Participant-Level Experimental Results

To make the participant-level results easier to read, the recorded data were organized into three complementary tables instead of one large table. Table 5 reports gaze calibration and validation outcomes, Table 6 reports BCI command and task-completion outcomes, and Table 7 reports Android execution and usability outcomes. This structure allows the reader to follow the results by system component: gaze mapping, BCI command control, and Android execution.

The participant-level results were compiled from the available backend telemetry, automatically generated task summaries, anonymous questionnaire responses, and the paper observation sheets completed during each session. When an automatically recorded event was unavailable, the corresponding value was recovered from the participant’s observation sheet and cross-checked against the remaining session records.

The participant-level data reported in Table 5, Table 6 and Table 7 provide the basis for the aggregated analysis of the proposed hybrid gaze–BCI interaction system. After completing the experimental sessions, the recorded values were used to calculate gaze validation performance, BCI command success, task timeout rate, Android execution reliability, and perceived usability. The following analysis focuses on aggregated indicators rather than on individual participant interpretation.

Table 5, Table 6 and Table 7 should be read together. The same anonymous participant codes are used in all three tables. For example, a participant with lower gaze-validation quality in Table 5 can be checked against command success and timeout values in Table 6, and then against execution and usability values in Table 7. The following subsections summarize these participant-level values into aggregated indicators and figures.

### 3.2. Aggregated Gaze Calibration and Validation Results

**Figure 6:**
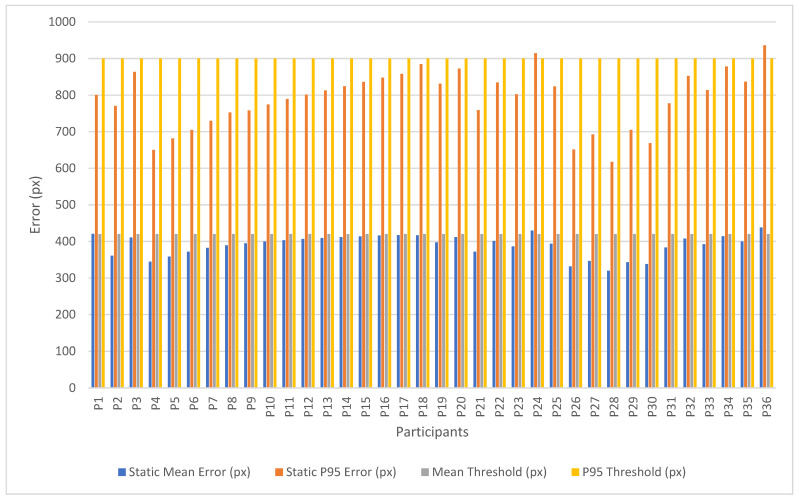
Distribution of static mean error and static p95 error across the 36 participants.

The gaze calibration and validation outcomes were aggregated across the 36 participants (see Figure 6). The analysis considered the number of participants who required recalibration, the number of promoted mappings, the static mean error, and the static p95 error. These indicators were used to evaluate whether the gaze-to-screen mapping provided a stable basis for the live Patient Assist task.

Across the 36 participants, 23 participants required at least one recalibration step, while 33 mappings were promoted to the active profile. The average static mean error was 390.02 px, and the average static p95 error was 789.09 px. Three participants completed the live task using the latest session-local mapping and the available execution fallback mechanisms.

### 3.3. BCI Command and Task Completion Results

**Figure 7:**
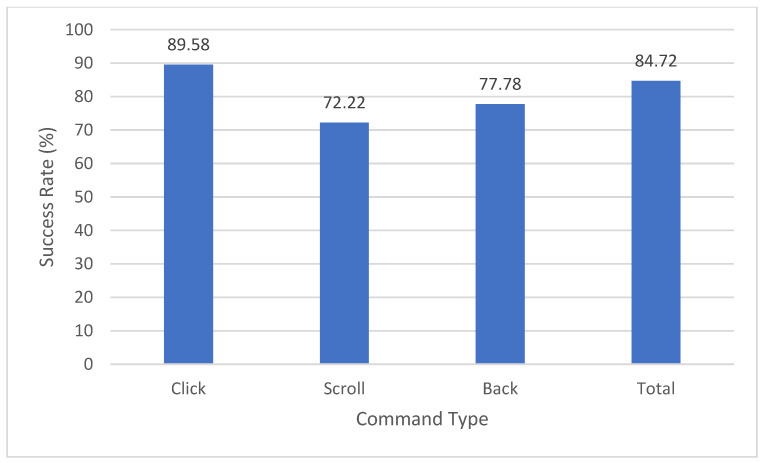
Command-specific success rates for click, scroll, and back actions.

The BCI command results were analyzed separately for click, scroll, and back actions. Since each participant completed four click windows, one scroll window, and one back window, the complete dataset included 144 expected click commands, 36 expected scroll commands, and 36 expected back commands (see Figure 7). The command-specific success rates were calculated using Equation (5), while timeout events were calculated using Equation (6).

The command-specific results showed 129 successful click commands out of 144 expected click windows, corresponding to 89.58%. Scroll commands were successful in 26 out of 36 expected windows, corresponding to 72.22%, while back commands were successful in 28 out of 36 expected windows, corresponding to 77.78%. Overall, 183 out of 216 expected BCI command windows were completed successfully, corresponding to 84.72%.

Across the standardized live task, the maximum allowed duration for the full button sequence was 160 s. However, this was only the upper limit, because the system moved to the next step as soon as the expected gaze fixation or BCI command was detected. The recorded task durations ranged from 83 s to 142 s, with all participants remaining below the maximum allowed duration. Across the complete task, 60 timeout steps were recorded out of 360 expected steps, corresponding to a timeout rate of 16.67%. Therefore, 300 out of 360 task steps were completed within the allowed time windows.

### 3.4. Android Execution and Interaction Reliability

**Figure 8:**
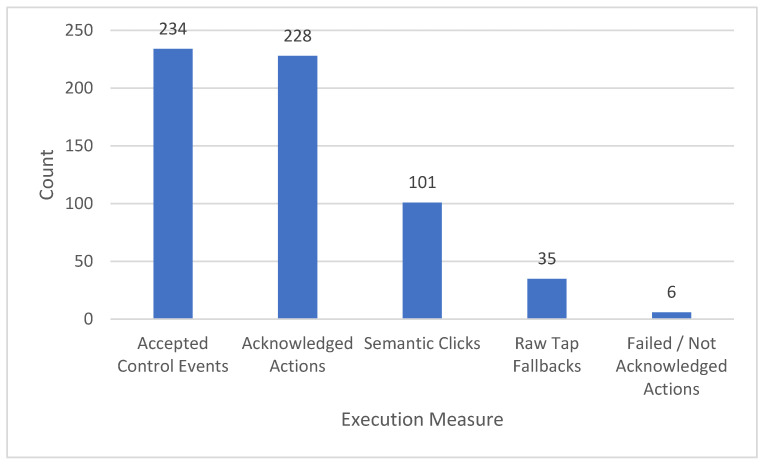
Android execution outcomes, including acknowledged actions, semantic clicks, and raw tap fallback events.

The Android execution layer was evaluated using accepted control events, acknowledged actions, semantic clicks, and raw tap fallback actions. These indicators were used to determine how often accepted BCI commands were converted into completed smartphone actions and how often the system relied on semantic Accessibility execution instead of fallback tapping (see Figure 8).

The Android execution layer received 234 accepted control events, of which 228 were acknowledged by the Android application. This corresponds to an acknowledgement rate of 97.44%. For click-type executions, 101 actions were completed through semantic Accessibility clicks, while 35 actions used raw tap fallback.

During the standardized Patient Assist task, fallback actions were counted as successful only when the intended large interface tile or expected action was completed and acknowledged by the Android application. No separate accidental-activation category was recorded during this feasibility test. Therefore, the reported fallback count shows how often the system needed coordinate-based tapping, but it does not provide a separate safety analysis of unintended fallback actions.

For the gaze-based target-selection windows, the participants correctly selected 111 targets out of 144 expected gaze-fixation targets, corresponding to 77.08%. This value describes the operational precision of the simplified Patient Assist sequence in the tested setup. The result should be interpreted together with the large-button interface design, because the tested task used preset assistive buttons and not small keyboard keys or dense smartphone menus.

### 3.5. Usability Results

**Figure 9:**
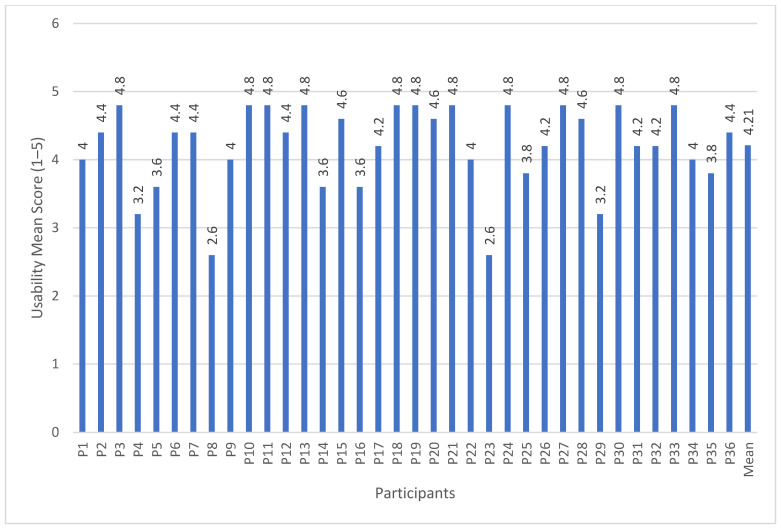
Mean usability scores reported by the participants after the hybrid gaze–BCI interaction task.

The usability results were calculated from the anonymous five-point Likert questionnaire completed after the live interaction task. The mean score was calculated for each participant and then aggregated across the full sample. These values were used to describe participant-perceived ease of use, comfort, gaze selection, BCI reliability, and general interest in hands-free smartphone interaction (see Figure 9).

The usability mean scores ranged from 2.6 to 4.8 on the five-point scale. The overall mean usability score was 4.21, indicating that most participants rated the interaction positively after completing the short live task.

The questionnaire included one item on perceived reliability of the BCI trigger, but it did not ask participants to rate click, scroll, and back commands separately. Therefore, the reported usability score should be interpreted as an overall perception of the short hybrid interaction task, not as a command-specific satisfaction measure. This is important because the measured BCI success rates differed across commands, with click reaching 89.58%, scroll 72.22%, and back 77.78%.

## 4. Discussion

The results show that the implemented system can support a short hybrid gaze–BCI smartphone interaction task in a controlled setting. The evaluation was not designed as a clinical validation and was not intended to compare the system directly with commercial accessibility tools. Instead, it tested whether the main implemented components could work together: mobile gaze tracking, gaze calibration and validation, BCI command filtering, WebSocket routing, Android Accessibility execution, and the simplified Patient Assist interface.

The gaze results indicate that the calibration and validation workflow provided usable mappings for most participants. Out of 36 participants, 33 mappings were promoted to the active profile. The average static mean error was 390.02 px, while the average static p95 error was 789.09 px. These values remained below the experimental acceptance thresholds of 420 px for mean error and 900 px for p95 error at group level. However, three participants did not obtain a promoted mapping and completed the task using the latest session-local mapping and the available fallback mechanisms. This shows that mobile camera-based gaze tracking can support the proposed interaction, but it also confirms that gaze calibration remains sensitive to user posture, phone position, lighting, and individual behaviour. This observation is consistent with previous work on gaze-based assistive interaction and smartphone-based eye tracking, where calibration and environmental conditions remain important factors [12,13,14,15].

The experimental threshold of 420 px was used as a profile-promotion criterion, not as a task-specific button-selection threshold. This means that a mapping above this value was not automatically excluded from the live task. In the Patient Assist interface, the buttons were large, and the Android execution layer also used semantic target selection and permitted raw tap fallback. This explains why some participants who did not obtain a promoted mapping were still able to complete the live sequence. However, this also shows that the static validation threshold and the live task requirement are not identical. A future version should define task-specific gaze thresholds based on the physical size, spacing, and layout of the interface tiles.

The operating dynamics of the current prototype should be understood in relation to the simplified Patient Assist interface. The tested sequence was not a random full smartphone navigation task. It was a controlled sequence of preset assistive buttons, with four gaze-fixation windows and six BCI command windows. In this setting, the maximum duration was 160 s, but the real durations ranged from 83 s to 142 s because successful actions moved the participant directly to the next step. The target-selection result was 111 correct targets out of 144 expected gaze targets, corresponding to 77.08%. This indicates that the system can support a short preset-button sequence, but it does not yet describe free typing, dense menu navigation, or long daily smartphone use.

The standardized Patient Assist sequence should therefore be interpreted as a short feasibility task, not as a simulation of full real-life smartphone use. The task did not include application switching, long menu navigation, unexpected notifications, communication interruptions, repeated correction attempts, or longer cognitive fatigue. These elements are common in daily smartphone use and may change both gaze stability and BCI command reliability. The present evaluation was limited to checking whether the main components could work together during a controlled assistive sequence. Longer and less structured tests are needed before drawing conclusions about daily use.

The present study reports gaze precision in screen pixels rather than in visual degrees. The static validation produced an average mean error of 390.02 px and an average p95 error of 789.09 px. A direct conversion to angular precision would require the physical pixel size of the screen and the exact eye-to-screen distance for each participant and session. These values were not recorded as separate experimental variables. For this reason, the current results should not be interpreted as a precise angular accuracy estimate. In practical terms, the prototype is suitable for large preset interface tiles, such as those used in the Patient Assist screen, but not yet for small buttons, keyboard keys, or dense smartphone menus. Future versions should measure eye-to-screen distance, report angular error, and define minimum safe target sizes for static and dynamic use.

Another limitation of the current gaze calibration is the use of a fixed Ridge regularization parameter. The same λ value was used for all participants and calibration sessions. This made the procedure consistent across the experiment, but it did not adapt the mapping to the number of calibration targets, user posture, lighting conditions, or device placement. A future version should test a small grid search or cross-validation step for each calibration session, so that the regularization strength can be selected based on the quality of the recorded calibration data.

The BCI command results were different across the three trained commands. Click commands reached 89.58% success, while scroll and back reached 72.22% and 77.78%, respectively. This result is reasonable because the click command was repeated four times in the live sequence and was linked to the most direct interaction goal. The scroll and back commands were tested only once per participant and may require more training or clearer mental separation. These results should not be read as proof that one command is generally better than another. They only show how the three commands behaved in this short task and with this specific training procedure. Previous BCI studies also report that EEG-based commands depend on training quality, signal stability, user attention, and the way the command is defined [4,5,6,7,8,9,10,11].

The present study did not analyze the neurophysiological separability of the click, scroll, and back mental commands. No confusion matrix, EEG feature-level analysis, or command-class separability analysis was included. Therefore, the reported command-specific values should be interpreted as functional success rates inside the implemented Emotiv Cortex workflow, not as proof that the three mental commands are neurophysiologically distinct. Future work should include command-level confusion matrices, repeated-session analysis, and EEG feature analysis to better understand how separable these commands are for each user.

The current BCI decision gate is also conservative because the consecutive counter is reset after any command sample below the confidence threshold. This behaviour reduces the risk of accidental activations, which is important in assistive interaction, but it may also reject valid commands when the signal contains short confidence drops. A leaky counter or a majority-vote window could be more tolerant to transient signal noise. Such a mechanism should be compared with the current rule in future tests, using both command success and accidental-trigger rate as evaluation measures.

The 22 min BCI training stage should also be considered a practical learning cost. In the present study, this duration was acceptable for a controlled laboratory feasibility session because each participant trained a neutral state and three active mental commands before the short live task. However, this duration may be too long for regular daily use, especially for users with fatigue, reduced attention, or limited tolerance for repeated training. A practical version of the system should reduce this cost by reusing stable user profiles, shortening the number of active commands, improving command training guidance, or adapting the profile over repeated sessions.

Some participant-level results also show the limits of the current prototype. P23 had only two correct BCI commands out of six, six timeout steps, the longest task duration of 142 s, and one of the lowest usability scores, 2.6 out of 5. This suggests that BCI command stability and task timing were difficult for this participant, even if the gaze mapping was promoted. P36 represents a different case. This participant did not obtain a promoted gaze mapping, with a static mean error of 438.2 px and a p95 error of 936.1 px, but completed all six BCI commands correctly and reported a usability score of 4.4. This shows that gaze mapping quality, BCI command success, and perceived usability do not always fail together. These cases support the need for adaptive calibration, better command training, and more flexible task-level monitoring.

The hybrid design helped separate target localization from action triggering. Gaze was used to identify the intended screen area, while BCI commands were used to start the action. This is close to the idea used in eye–brain interfaces, where gaze gives the spatial target and neural activity contributes to intentional selection [16,17]. Compared with these works, the present system focuses on an Android smartphone interface and uses Android Accessibility for execution. The contribution is therefore more practical and engineering-oriented: the system does not only detect gaze or neural commands, but also routes accepted commands to a mobile interface and records the execution result.

The Android execution results also support the feasibility of the implemented architecture. From 234 accepted control events, 228 were acknowledged by the Android application, giving an acknowledgement rate of 97.44%. This indicates that the backend-to-Android routing and acknowledgement mechanism worked in most cases during the test. At the same time, the use of 35 raw tap fallback actions shows that semantic Accessibility execution was not always possible. This is not unexpected, because Android interfaces may expose different accessibility nodes depending on the screen state, application layout, and timing. In the present task, fallback tapping was used only inside the simplified Patient Assist interface with large preset tiles. However, the study did not include a separate accidental-activation analysis for fallback events. This is an important limitation for assistive use, because coordinate-based tapping may become unsafe on small buttons or dense interfaces. For this reason, the fallback mechanism was useful as a practical support layer in the tested interface, but future versions should reduce fallback use and add explicit logging of correct fallback selections versus accidental activations.

The usability results were also positive in general. The average usability score was 4.21 out of 5, with participant-level means ranging from 2.6 to 4.8. This suggests that most participants found the interaction understandable and usable during the short session. Lower scores for some participants may be related to gaze calibration difficulty, BCI command training, headset comfort, or timeout events during the task. Since the participants were student volunteers and not clinical users, these usability results should be interpreted as early feedback on the prototype interface, not as evidence of medical suitability.

The aggregate usability score may also hide differences between command types. In the current questionnaire, perceived BCI reliability was collected as a single overall item after the complete task. Participants were not asked to rate click, scroll, and back reliability separately. This limits the interpretation of perceived reliability, because the measured command success rates were not identical across commands. A future questionnaire should include command-specific perceived reliability items, so that objective command success and subjective satisfaction can be compared for click, scroll, and back actions separately.

Compared with previous gesture-based or vision-based assistive systems, the proposed system uses a different type of input combination. Gesture-based systems can provide touchless control using camera input, but they still require visible hand or body movement [21,23]. The present system reduces the need for hand movement by using gaze and EEG-based commands. Compared with BCI-only systems, the proposed approach does not rely on neural commands to identify the screen target; instead, BCI is used mainly as an action trigger [7,10,11]. This reduces the complexity of the BCI command set, but it also introduces the need for a stable gaze pipeline. Therefore, the system should be understood as a hybrid alternative rather than as a replacement for gaze-only, voice-based, switch-based, or BCI-only approaches.

To make the comparison with related works easier to follow, Table 8 summarizes the relation between the present system and selected previous studies. The comparison is descriptive because the studies used different devices, tasks, users, and evaluation protocols.

Table 8 does not rank the systems, because the evaluation protocols are different. It shows that the present work is closer to an engineering integration study. The system combines gaze localization, EEG command triggering, Android execution, backend routing, and task-level logging in one smartphone-based prototype.

Future assistive smartphone interfaces may also benefit from models that use previous movement or interaction patterns to estimate the next likely action. Meyer et al. showed that stochastic models can be used to study movement prediction and to estimate future motion from previous movement patterns [27]. In a later version of the proposed system, a similar idea could be useful for gaze-based smartphone control. For example, if a user wants to type on a smartphone or tablet, the system could combine gaze direction, previous selections, interface context, and language prediction to reduce the number of required actions. This function is not implemented in the current prototype. The present system only supports preset Patient Assist buttons and simple click, scroll, and back commands. Future work should therefore include gaze-path prediction, process prediction, and context-based word suggestions for faster text entry.

### 4.1. Limitations

The study has several limitations. First, the evaluation used 36 student volunteers, not users with motor impairments. Second, the live task was short, lasted a maximum of 160 s, and did not simulate full real-life smartphone use, including application switching, interruptions, long navigation paths, or cognitive fatigue. Third, gaze precision was reported in screen pixels and not in visual degrees, because eye-to-screen distance and physical pixel size were not recorded as independent experimental variables. The gaze acceptance threshold was not defined directly from Patient Assist tile size, spacing, or layout. The gaze-mapping model also used a fixed Ridge regularization parameter, without per-session grid search or cross-validation. Fourth, the system was tested in controlled indoor conditions, with stable lighting and a fixed phone position. Fifth, the results depend on the Emotiv headset, the trained mental commands, and the Android device used in the prototype. Sixth, the study did not include a direct comparison with gaze-only control, voice access, switch scanning, or other Android Accessibility methods.

The study also did not include confusion matrices or EEG feature-level analysis for the three mental commands. The BCI decision gate also used a strict consecutive-detection rule, which may reject valid commands affected by short confidence drops. The 22 min BCI training stage also represents a practical learning cost and may be too long for daily use.

The fallback mechanism was not analyzed with a separate category for correct fallback selections versus accidental fallback activations.

Some participant-level cases also showed that gaze quality, BCI command success, timeout behaviour, and usability may fail in different ways and should be analyzed separately in future tests.

The usability questionnaire also did not collect command-specific perceived reliability scores for click, scroll, and back.

For these reasons, the results should be considered as a feasibility evaluation of the implemented architecture.

### 4.2. Future Work

Future work should include longer interaction sessions, tests on more smartphone models, and a direct comparison with gaze-only and other accessibility workflows. It would also be useful to test more robust gaze models, per-session Ridge parameter selection, adaptive BCI thresholds, leaky-counter or majority-vote BCI decision gates, shorter BCI training workflows, profile reuse across sessions, improved semantic target selection, explicit fallback safety logging, reduced raw tap fallback use, command-specific usability and perceived-reliability items, angular-error reporting, task-specific gaze thresholds based on tile size, and minimum safe button-size estimation. A later study should include users with real assistive needs, but only after the technical workflow is stable enough for longer and safer use.

## 5. Conclusions

This paper presented an implemented hybrid gaze–BCI system for assistive smartphone interaction. The system combines mobile gaze tracking, gaze calibration, EEG-based command triggering, FastAPI backend routing, Android Accessibility execution, and a simplified Patient Assist application.

The evaluation with 36 student volunteers showed that the main system components worked together during a short controlled task. Most gaze mappings were promoted to the active profile, and the average static validation errors remained below the selected experimental thresholds. The BCI command results showed different success levels for click, scroll, and back actions, with click commands having the highest success rate in this protocol. The Android execution layer also showed a high acknowledgement rate, while the raw tap fallback remained useful when semantic target execution was not available.

The usability scores suggest that most participants found the system understandable and usable during the test. Still, the results should be read as an early feasibility evaluation, not as a clinical validation. The study used student volunteers, a controlled indoor setup, and a short interaction sequence.

Future work should test the system in longer sessions, on more smartphone models, and with users who have real assistive needs. Further improvements should also focus on more stable gaze calibration, better BCI command separation, and stronger semantic target selection inside Android interfaces.

## Figures and Tables

**Table 1:** Internal gaze-mapping quality thresholds and experimental live-interaction acceptance thresholds.

Quality Measure	Soft Mapping Threshold	Hard Rejection Threshold	Experimental Live-Interaction Acceptance Threshold
Mean error	≤320 px	≤580 px	≤420 px
P95 error	≤700 px	≤1200 px	≤900 px
Horizontal coverage	≥0.78	≥0.72	—
Vertical coverage	≥0.82	≥0.70	—
Horizontal correlation	≥0.78	≥0.72	—
Vertical correlation	≥0.78	≥0.63	—
Horizontal cross-talk	≤0.40	≤0.55	—
Minimum target count	—	—	25

**Table 2:** Testing procedure completed by each participant.

Stage	Procedure	Duration
1	System presentation, informed consent, and anonymous participant code assignment	5 min
2	Smartphone positioning, lighting check, Android permissions, and foreground service activation	4 min
3	Anonymous profile selection or creation	2 min
4	Gaze calibration using 9–25 sequential screen targets	4 min
5	Static baseline validation using a 25-point grid	3 min
6	Rotate Phone validation and mapping promotion decision	3 min
7	Short Patient Assist gaze-only tile verification	2 min
8	EEG headset placement and signal-quality verification	5 min
9	Training of neutral state and active mental commands for click, scroll, and back	22 min
10	Standardized live Patient Assist task	2 min 40 s
11	Anonymous usability questionnaire	4 min
	Total session duration	approximately 56 min

**Table 3:** Standardized live Patient Assist task and maximum time windows used for each participant.

Step	Interface State/Target	Participant Action	Maximum Duration
1	Patient App open; Needs tile visible	Fix gaze on Needs	up to 10 s
2	Needs area	Imagine click action	up to 20 s
3	Needs area	Imagine scroll action	up to 20 s
4	Tired option visible	Fix gaze on Tired	up to 10 s
5	Tired option	Imagine click action	up to 20 s
6	Needs screen/navigation state	Imagine back action	up to 20 s
7	Home screen; Help tile visible	Fix gaze on Help	up to 10 s
8	Help area	Imagine click action	up to 20 s
9	Call Caregiver option visible	Fix gaze on Call Caregiver	up to 10 s
10	Call Caregiver option	Imagine click action	up to 20 s
	Total		160 s

**Table 4:** Measures used to evaluate the implemented hybrid gaze–BCI system.

System Component	Evaluation Measure	Description
Gaze calibration and validation	Validation mean error	Average distance between displayed validation target and estimated gaze point
Gaze calibration and validation	Validation p95 error	Error value below which 95% of recorded validation errors were located
Gaze calibration and validation	Mapping acceptance status	Whether the validated mapping was promoted to the active profile
BCI command layer	Accepted neural triggers	Mental commands that passed the decision gate
BCI command layer	Rejected command events	Events rejected because of confidence, consecutiveness, or cooldown conditions
BCI command layer	Click command success rate	Percentage of expected click command periods correctly routed as click events
BCI command layer	Scroll command success rate	Percentage of expected scroll command periods correctly routed as scroll events
BCI command layer	Back command success rate	Percentage of expected back command periods correctly routed as back events
Complete interaction loop	Full sequence completion rate	Percentage of participants who completed the full 160 s sequence without critical interruption
Complete interaction loop	Target-selection accuracy	Percentage of requested targets correctly activated
Android execution layer	Successful action-execution rate	Percentage of accepted control events completed and acknowledged
Android execution layer	Semantic-click execution rate	Percentage of completed actions executed on semantic accessibility targets
Android execution layer	Raw tap fallback rate	Percentage of completed actions executed through direct tap fallback
Complete interaction loop	Command-response latency	Time from accepted BCI trigger to Android acknowledgement for command steps, and time from fixation-window start to confirmation for gaze-fixation steps
Standardized live task	Timeout rate	Percentage of gaze-fixation or BCI command steps not completed within the maximum allowed time window
Standardized live task	Actual task duration	Time required by a participant to complete the live sequence, with 160 s as the maximum duration
User questionnaire	Perceived usability scores	Likert-scale responses concerning ease, comfort, reliability, and interest

**Table 5:** Participant-level calibration and validation results.

Participant	Calibration Targets	Recalibration	Static Mean Error (px)	Static P95 Error (px)	Rotate Validation	Mapping Promoted
P1	16	YES	420.4	800.2	Completed	NO
P2	16	YES	361.2	770.6	Completed	YES
P3	16	NO	410.7	863.5	Completed	YES
P4	16	YES	344.6	650.4	Completed	YES
P5	16	YES	358.9	681.7	Completed	YES
P6	16	NO	371.9	704.9	Completed	YES
P7	16	YES	382.7	729.5	Completed	YES
P8	16	YES	389.5	752.6	Completed	YES
P9	16	NO	395.2	758.1	Completed	YES
P10	16	NO	399.8	774.3	Completed	YES
P11	16	YES	403.6	789.6	Completed	YES
P12	16	NO	406.9	801.4	Completed	YES
P13	16	YES	409.5	812.5	Completed	YES
P14	16	YES	411.8	824.1	Completed	YES
P15	16	YES	414.1	836.2	Completed	YES
P16	16	YES	416.2	847.5	Completed	YES
P17	16	YES	417.3	858.1	Completed	YES
P18	16	YES	416.8	884.6	Completed	YES
P19	16	YES	397.5	831.3	Completed	YES
P20	16	YES	411.8	872.6	Completed	YES
P21	16	NO	372.2	758.8	Completed	YES
P22	16	YES	401.5	834.6	Completed	YES
P23	16	YES	386.6	802.1	Completed	YES
P24	16	NO	429.4	914.8	Completed	NO
P25	16	NO	394.2	823.7	Completed	YES
P26	16	NO	331.8	651.4	Completed	YES
P27	16	YES	346.9	692.2	Completed	YES
P28	16	NO	319.7	617.5	Completed	YES
P29	16	YES	343.4	704.9	Completed	YES
P30	16	YES	338.2	668.6	Completed	YES
P31	16	YES	383.6	777.3	Completed	YES
P32	16	NO	407.9	852.5	Completed	YES
P33	16	NO	392.5	813.9	Completed	YES
P34	16	YES	414.5	878.4	Completed	YES
P35	16	NO	399.8	836.7	Completed	YES
P36	16	YES	438.2	936.1	Completed	NO

Note: Participants with mappings that were not promoted completed the live task using their latest session-local mapping together with the available execution fallback mechanisms.

**Table 6:** Participant-level BCI and task results.

Participant	Click Success (0–4)	Scroll Success (0–1)	Back Success (0–1)	Correct Commands (0–6)	Timeout Steps (0–10)	Actual Task Duration (s)
P1	3	0	1	4	2	103
P2	4	1	0	5	1	95
P3	4	1	1	6	0	90
P4	2	0	1	3	4	113
P5	4	0	1	5	2	124
P6	4	1	0	5	2	111
P7	4	1	0	5	1	88
P8	2	0	0	2	5	121
P9	3	1	1	5	3	118
P10	4	1	1	6	0	87
P11	4	1	1	6	0	84
P12	4	0	1	5	2	105
P13	4	1	1	6	0	89
P14	3	0	1	4	3	118
P15	4	1	1	6	1	91
P16	3	1	0	4	3	125
P17	3	1	1	5	1	101
P18	4	1	1	6	0	86
P19	4	1	1	6	0	92
P20	4	1	1	6	1	96
P21	4	1	1	6	0	88
P22	3	1	1	5	2	107
P23	2	0	0	2	6	142
P24	4	1	1	6	0	83
P25	3	1	1	5	2	112
P26	3	1	1	5	2	109
P27	4	1	1	6	1	99
P28	4	0	1	5	1	86
P29	4	0	0	4	4	131
P30	4	1	0	5	1	90
P31	4	1	1	6	2	108
P32	4	1	1	6	3	124
P33	4	1	1	6	0	89
P34	3	1	1	5	1	97
P35	4	0	1	5	2	139
P36	4	1	1	6	2	116

**Table 7:** Participant-level execution and usability results.

Participant	Correct Targets	Accepted Control Events	Acknowledged Actions	Semantic Clicks	Raw Tap Fallbacks	Usability Mean
P1	3	6	6	2	1	4.0
P2	4	6	5	3	1	4.4
P3	4	6	6	4	0	4.8
P4	3	7	6	2	1	3.2
P5	3	8	8	3	1	3.6
P6	3	6	5	4	0	4.4
P7	4	7	7	3	1	4.4
P8	3	7	7	2	1	2.6
P9	2	7	7	3	0	4.0
P10	4	7	6	2	2	4.8
P11	4	6	6	3	1	4.8
P12	3	7	7	3	1	4.4
P13	4	6	6	3	1	4.8
P14	3	8	7	2	1	3.6
P15	3	6	6	2	2	4.6
P16	3	7	7	3	0	3.6
P17	3	6	6	1	2	4.2
P18	3	6	6	3	1	4.8
P19	4	6	6	3	1	4.8
P20	3	7	7	3	1	4.6
P21	4	6	6	3	1	4.8
P22	3	6	6	3	1	4.0
P23	2	8	7	2	0	2.6
P24	3	6	6	3	1	4.8
P25	3	6	6	2	2	3.8
P26	3	6	6	4	0	4.2
P27	3	6	6	4	0	4.8
P28	4	7	7	4	0	4.6
P29	2	7	7	3	2	3.2
P30	4	6	6	4	0	4.8
P31	2	6	6	3	1	4.2
P32	1	6	6	3	2	4.2
P33	3	6	6	4	0	4.8
P34	3	7	7	0	3	4.0
P35	3	7	7	3	1	3.8
P36	2	6	6	2	2	4.4

**Table 8:** Descriptive comparison with selected related works.

Study	Main Input	Target Application	Reported Evaluation	Relation to the Present Study
Drăgoi et al. [10]	EEG-based BCI	Home automation	Real-time BCI-based device control	Uses BCI commands for external control, but not gaze-based smartphone target selection
Reddy et al. [16]	Gaze and neural signal	XR target selection	Eye–brain interface concept for target selection	Similar separation between gaze target and neural confirmation, but in XR rather than Android
Coutray et al. [17]	EEG and eye tracking	VR hands-free interaction	Hybrid EEG and eye-tracking interaction	Similar hybrid input idea, but tested in virtual reality
Zahra et al. [21]	Camera-based hand gestures	Interactive digital display	Vision-based gesture interaction	Supports hands-free interaction, but still requires visible hand movement
Phuong and Cong [23]	Vision-based hand tracking	Robot arm control	Camera-based hand tracking for teleoperation	Uses camera-only human tracking, but not EEG or smartphone Accessibility
Present study	Gaze and EEG BCI	Assistive smartphone interaction	36 participants; 33/36 mappings promoted; BCI success: 89.58% click, 72.22% scroll, 77.78% back; Android acknowledgement: 97.44%; usability: 4.21/5	Integrates gaze localization, EEG command triggering, Android Accessibility execution, backend routing, and Patient Assist task

## Data Availability

The original contributions presented in this study are included in the article. Further inquiries can be directed to the corresponding author.

## Appendix A. Algorithmic Details of the Implemented Control Workflow

**Algorithm A1**. Gaze calibration, validation, and profile promotion
1: FUNCTION RunGazeCalibration(activeProfile, activeDevice) 2: Display calibration targets on the smartphone screen 3: FOR each target position DO 4: Capture frames and facial landmarks 5: Run TinyTrackerS model 6: Store model output and known target coordinate 7: END FOR 8: Group recorded samples by target 9: Remove invalid samples and reduce extreme values 10: Build polynomial feature vectors 11: Fit horizontal mapping coefficients using Ridge regression 12: Fit vertical mapping coefficients using Ridge regression 13: Compute fitting quality indicators 14: IF hard rejection thresholds are exceeded THEN 15: Reject calibration 16: Display retry message 17: RETURN 18: END IF 19: Display validation targets 20: Record validation gaze points 21: Compute mean error, p95 error, coverage, correlation, and cross-talk 22: IF validation thresholds are satisfied THEN 23: Save mapping to active profile 24: Update mapping priors 25: Display calibration success message 26: ELSE 27: Keep the previous active mapping 28: Store validation failure 29: Display retry message 30: END IF 31: END FUNCTION

**Algorithm A2**. BCI connection and neural command decision gate
1: FUNCTION StartBCISession(activeProfile) 2: Receive connection request from Android interface 3: Start Cortex service 4: Request access to Emotiv runtime 5: Authorize the Cortex session 6: Connect the EEG headset 7: Load the trained mental command profile 8: Subscribe to the mental command stream 9: Notify the Android interface that BCI is ready 10: WHILE the session is active DO 11: Receive raw mental command and confidence value 12: IF command confidence < 0.60 THEN 13: Reset consecutive command counter 14: CONTINUE 15: END IF 16: Increase consecutive command counter 17: IF consecutive command counter < 3 THEN 18: CONTINUE 19: END IF 20: IF the cooldown interval is still active THEN 21: CONTINUE 22: END IF 23: Map the accepted mental command to click, scroll, or back 24: Create the corresponding control event 25: Publish the control event through the FastAPI control endpoint 26: Reset consecutive command counter 27: Start cooldown interval 28: END WHILE 29: END FUNCTION

**Algorithm A3**. Android Accessibility target selection and control execution
1: FUNCTION ExecuteControlEvent(controlEvent, currentGazePoint) 2: Check event_id 3: IF event_id was already processed THEN 4: Ignore duplicated event 5: RETURN duplicate acknowledgement 6: END IF 7: Log the incoming control event 8: Check cached clickable targets 9: IF valid cached targets are unavailable THEN 10: Query visible Android Accessibility nodes 11: Store clickable candidate targets 12: END IF 13: FOR each candidate target DO 14: Compute whether gaze point is inside target bounds 15: Compute distance to target edge 16: Compute distance to target centre 17: Calculate candidate relevance score 18: END FOR 19: Select the most probable stable target 20: IF a semantic target is available THEN 21: Execute Accessibility action click 22: Set execution status to semantic click executed 23: ELSE IF raw tap fallback is enabled THEN 24: Execute tap at current gaze coordinates 25: Set execution status to raw tap executed 26: ELSE 27: Set execution status to target not found 28: END IF 29: Update event cache 30: Send acknowledgement and execution status to backend 31: END FUNCTION
